# Locally Advanced Adenoid Cystic Carcinoma of the Breast—A Case Report with a Review of the Literature

**DOI:** 10.3390/medicina59112005

**Published:** 2023-11-15

**Authors:** Joanna Rypel, Paulina Kubacka, Joanna Mykała-Cieśla, Jacek Pająk, Weronika Bulska-Będkowska, Jerzy Chudek

**Affiliations:** 1 Department of Internal Medicine and Oncological Chemotherapy, Medical University of Silesia in Katowice, 40-055 Katowice, Poland; paulina.kubacka14@gmail.com (P.K.); jmyka@interia.pl (J.M.-C.); lek.weronikabulska@gmail.com (W.B.-B.); 2 Department of Pathomorphology, Medical University of Silesia in Katowice, 40-055 Katowice, Poland; jacek.pajak@sum.edu.pl

**Keywords:** triple-negative breast cancer, adenoid cystic carcinoma, metastasis, systemic therapy

## Abstract

Breast cancer (BC) is a heterogeneous disease distinguished by four main subtypes based on the expression of estrogen, progesterone receptors, and human epidermal growth factor-2 on the cancer cells. Triple-negative breast cancer (TNBC) consists of approximately 10–20% of all BCs and is characterized by a poor prognosis. Adenoid cystic carcinoma (ACC) of the breast is a rare, special type of TNBC with low metastatic potential and usually favorable prognosis. There are no established recommendations concerning systemic therapy in advanced ACC. We present a case of a 70-year-old woman with locally advanced ACC with progression after radical mastectomy, and review the literature concerning the treatment of metastatic disease focused on systemic therapy.

## 1. Introduction

According to the latest statistics of the GLOBCAN 2020, there were almost 19.3 million new cancer cases worldwide, and breast cancers accounted for 11.7% of them [1]. Breast cancer is a heterogeneous disease and is categorized into four major subtypes based on the expression of estrogen (ER) and progesterone (PR) receptors, human epidermal growth factor receptor-2 (HER2), cell proliferation marker Ki67: luminal A and B, HER-2 positive and triple-negative breast cancer (TNBC) [2]. TNBC does not express ER, PR, or HER2 and presents aggressive behavior, high invasiveness, early relapse, and poor prognosis [3]. It consists of approximately 10–20% of all breast cancers [4]. Immunohistochemically, TNBCs are grouped into two categories based on the expression of cytokeratins 5/6: basal and non-basal TNBC [5]. Burstein et al. identified four molecularly distinct TNBC subtypes: basal-like immune-activated (BLIA), basal-like immune-suppressed (BLIS), mesenchymal (MES), and luminal androgen receptor (LAR) [6].

Adenoid cystic carcinoma (ACC) of the breast belongs to a rare, special type of TNBC (basal subtype). This cancer has favorable prognosis related to rare regional lymph node involvement and metastases [7], in contrast to the poor outcome of most TNBC phenotypes. Three different histologic types of ACC are known: classic, solid basaloid, and subtype with high-grade transformation. Most of these tumors show fusion of MYB and NFIB genes and overexpression of MYB. ACC with adverse features may develop metastasis [8].

Due to the lack of the expression of ER and PR, and overexpression of HER2, there is no molecular targeted therapy in either radical or palliative settings. Furthermore, there are no established recommendations concerning systemic therapy in advanced cases, or after the progression behind metastasectomy and radiation therapy in oligometastatic dissemination of ACC of the breast. The rarity of the disease causes difficulties in the choice of the first and subsequent palliative lines of systemic therapies.

## 2. Materials and Methods

The patient was treated in the Department of Internal Diseases and Oncological Chemotherapy at the Medical University of Silesia in Katowice from December 2019 to May 2022. The patient had undergone a physical examination and assessment of laboratory parameters at every visit. After metastasectomy, the patient was actively followed up with imaging examinations performed every 6 months.

The surgically resected specimens were fixed in 10% neutral-buffered formalin and paraffin-embedded. Sections were stained with hematoxylin-eosin (HE). Immunohistochemistry (IHC) was performed on a BenchMark Ultra autostainer (Roche) and Linik48, Omnis Autostainer (DAKO). The monoclonal antibodies were used for CKAE1/AE3 (DAKO, GA 053), vimentin (DAKO, GA 630), CK7 (DAKO, GA 619), p63 (DAKO, GA 662), SMA (DAKO, GA 611), CK5/6 (DAKO, GA 780), CEA (DAKO, GA 622), CK19 (DAKO, GA 615), CK18 (DAKO, GA 618), p53 (DAKO, GA 616), CD117 (DAKO, A 4502), ER (DAKO, GA084), PR (DAKO, GA 090), Ki67 (DAKO, GA 626), CD10 (DAKO, GA 786), AMARC (DAKO, GA 060), HER-2 (Roche, 05278368001), GATA-3 (Roche, 07107749001), Calponin (Roche, 05435684001), PAX-8 (Roche, 06523927001), ABPAS (Bio-Optica Milano, 04-163802).

We identified 16 papers concerning descriptions of the treatment of metastatic cases of ACC of the breast in the literature that were included in this systematic review.

## 3. Case Report

**Figure 1:**
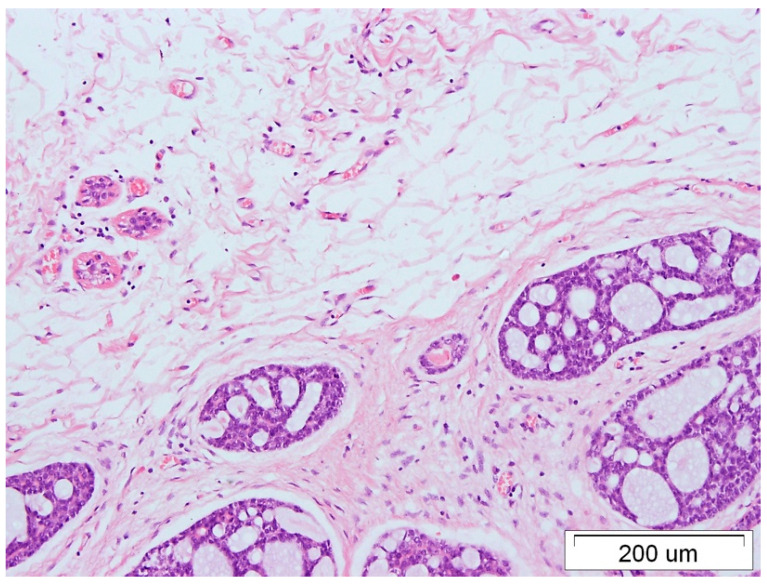
ACC of the breast, HE staining, 100× magnification.

**Figure 2:**
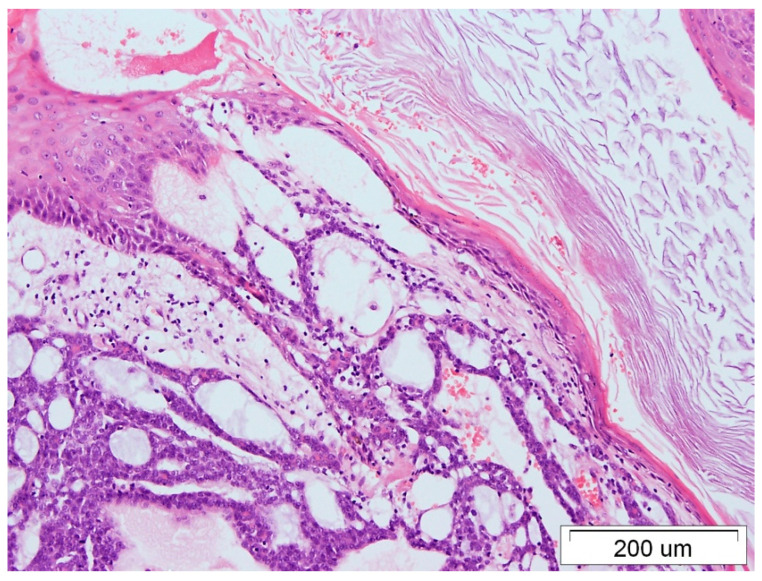
ACC of the breast with skin infiltration, HE staining, 100× magnification.

A 70-year-old woman presented with a mass in her left breast, which was ulcerated, and centrally located, and the papilla was not visible. After an oncological work-up, the patient was diagnosed with locally advanced TNBC cT4bN0M0, ACC subtype. The patient underwent radical mastectomy with a sentinel node biopsy (December 2017) and subsequent radiation therapy (February 2018). The pathology report demonstrated a mass measuring 6 × 5.5 × 5 cm, infiltrating the dermis, minor superficial ulcerations without stromal inflammation, no blockages in the vascular lumen, and confirmed a lack of lymphatic node involvement by the cancer cells (Figure 1 and Figure 2). Immunohistochemical examination on the pathological sections revealed no expression of hormone receptors and HER2, but positive for p63, CK5/6, and Ki67 15% (basal-like TNBC subtype).

**Figure 3:**
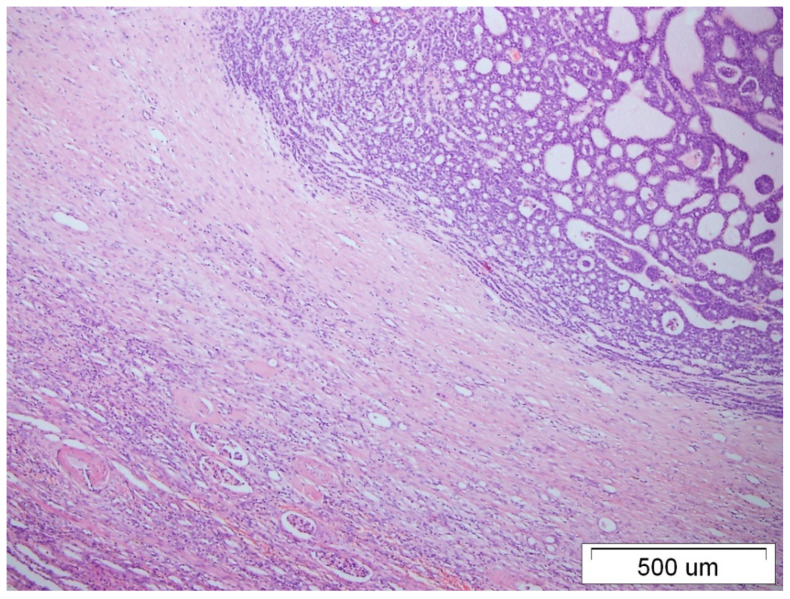
Renal tissue with metastatic ACC, HE staining, 40× magnification.

**Figure 4:**
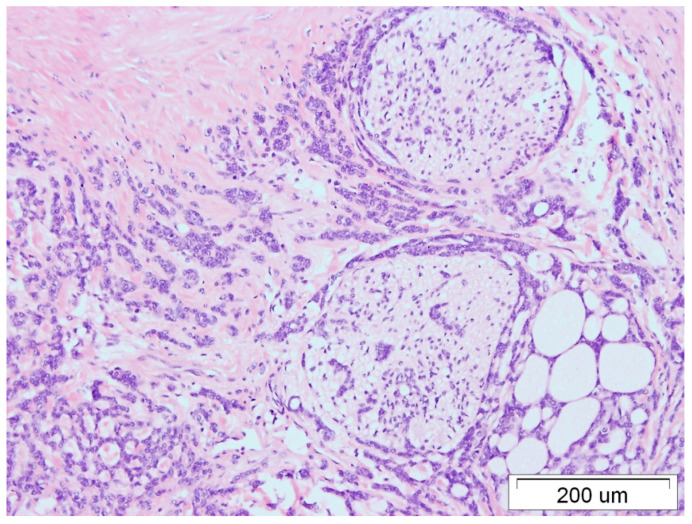
This infiltration on the nerve trunks of the kidney by ACC, HE staining, 100× magnification.

Two years later, due to pain, the patient underwent unscheduled abdominal ultrasound, which revealed a pathological mass in the left kidney. An urgently performed computed tomography (CT) scan confirmed a tumor of the left kidney with features of infiltration of the renal vein, measuring 70 × 60 × 56 mm. A radical nephrectomy was performed (December 2019). In the histological examination (pT4N0), nerve infiltrations with angioinvasion, as well as infiltration of the vascular pedicle and perirenal adipose tissue, were described (Figure 3 and Figure 4). The immunohistochemical examination confirmed a similar tumor morphology and immunostaining profile of the kidney and breast lesions (Table 1).

**Figure 5:**
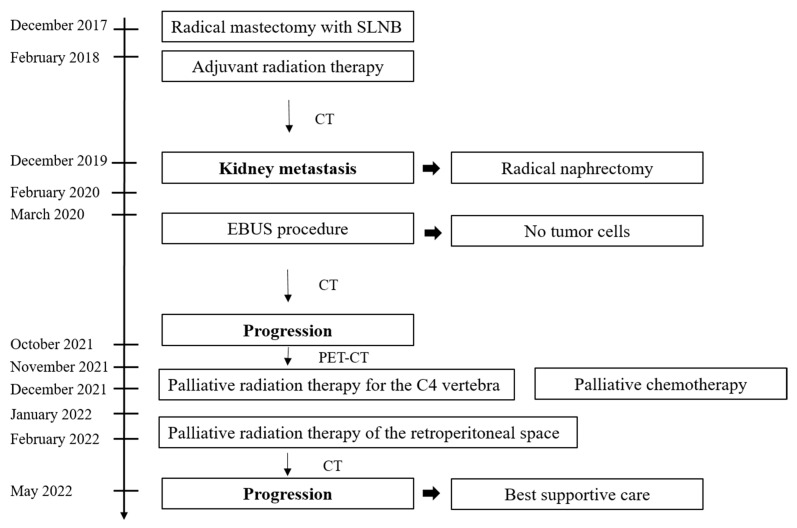
The timeline of events and interventions in the patient with breast ACC. CT—computed tomography, EBUS—endobronchial ultrasound, SLNB—sentinel node biopsy, PET-CT—positron emission tomography-computed tomography.

During the postsurgical follow-up, a PET-CT scan (February 2020) revealed metabolically active mediastinal lymph nodes, but no tumor cells were found in the cytological examination after the EBUS procedure (March 2020). A control chest CT scan after 3 months showed a small nodule in the left lung (less than 1 cm) and stable, similarly enlarged lymph nodes in the mediastinum. The next CT scan, after 6 months, showed progression of the lesion in the left lung (0.9 cm—previously 0.6 cm), a new nodule of 0.8 cm in the left lung. In October 2021, the patient was admitted to the hospital for 3 months for diagnosis of abdominal pain. CT imaging of the abdominal cavity showed infiltration in the post-nephrectomy location (numerous nodules) and spread to the lymph nodes in the abdominal cavity and chest, spleen (two lesions), right kidney (three hypodense lesions 10–14 mm in diameter), metabolically active in PET-CT (November 2021). In addition, the PET-CT revealed metastasis in the body of C_4_ vertebra. The patient was qualified for palliative radiation therapy for the C_4_ vertebra lesion and subsequent palliative chemotherapy (CTH) with doxorubicin and cyclophosphamide (December 2021). After two administrations the treatment was discontinued, due to toxicity with deterioration of the general clinical condition and further disease progression. Palliative radiation therapy (RTH) of the retroperitoneal space was administered, due to complaints of abdominal pain (January/February 2022). Two months after palliative RTH, the general condition gradually deteriorated. Progressive cancer cachexia and abdominal pain were observed. A control CT scan showed multiple nodules in both lungs, progression in the spleen, and post-nephrectomy location with aortic infiltration. Best supportive care was recommended as the only therapy (May 2022) (Figure 5).

## 4. Discussion

Adenoid cystic carcinomas have been reported in salivary glands, the external auditory canal, and other regions of the head and neck, digestive system, uterus, cervix, skin, prostate gland, and breast [9,10,11,12]. Breast ACC is a distinct and rare subtype of mammary malignancy, accounting for less than 0.1% of all breast cancers [13]. Generally, ACC exhibits a relatively indolent clinical course, characterized by a low propensity for lymph node involvement and distant metastases, with an excellent prognosis in most cases [14]. This case report presents the clinical course of the locally advanced disease with progression during the observation. The challenges associated with the management of metastatic ACC are the main points of this discussion, stressing the paucity of established treatment regimens.

In an attempt to provide the best perspective on the management of ACC, we present a summary of the published data, including case reports describing the stage of cancer at diagnosis, primary treatment, time to progression, site of metastasis, and secondary treatment with the obtained response. The primary treatments included mastectomy or lumpectomy with adjuvant RTH and CTH. However, the role of neoadjuvant/adjuvant CTH in ACC patients, even with axillary lymph involvement, remains unproven. Grabenstetter et al. documented a positive response to neoadjuvant CTH in a patient with solid-basaloid variant ACC [15]. On the other hand, the propensity score matched cohort by Yang et al. inferred that adjuvant CTH did not significantly improve OS in ACC patients [16].

The time to progression in aggressive cases varied widely, emphasizing the unpredictable nature of ACC’s clinical course, whereas, metastasis sites were quite typical for breast cancers (lungs, bones, liver, brain), except for the kidneys. The treatment after progression largely depended on the site and metastasis extent. Metastasectomy, as in our case, was utilized in oligometastatic disease, with an excellent prognosis in some cases. However, our patient developed systemically metastasized disease approximately one year after kidney metastasectomy.

In non-oligometastatic disease, doxorubicin- or taxane-based CTH was typically applied, resulting in SD or PD (Table 2). We qualified the patient for combined CTH due to the rapidly progressing disease with symptomatic metastases. The choice of doxorubicin and cyclophosphamide-based CTH was to obtain a rapid reduction of the cancer burden. Unfortunately, our patient developed significant toxicity after two AC cycles, necessitating treatment cessation, which precluded the assessment of therapy effectiveness. It is well known, that topoisomerase IIα is the main target of anthracyclines, which bind to this enzyme and lead to irreversible DNA damage [17]. Of note, Vranic S et al. reported low expression of topoisomerase IIα in the ACC tissue of the breast [18]. That raises doubts concerning the benefit of the anthracycline-based CTH. We did not determine the expression of this protein; however, no overexpression of epidermal growth factor receptor-1 (HER1), which plays a role in the stimulation of Ras/mitogen–activated protein kinase, the phosphoinositide-3-kinase/Akt, and the phospholipase-Cγ/protein kinase C pathways was reported in the literature. Potentially, inhibition of EGFR could represent an emerging target in advanced ACC of the breast.

This heterogeneous behavior of the disease is aligned with the findings of Xi et al., demonstrating that patients with ACC can present a spectrum of clinical manifestations, some of which are parallel with the aggressive course of the disease, typical for the TNBC phenotype [18]. In these cases, CTH is still a mainstay treatment. This may shortly change, as recently sacituzumab govitecan, a Trop-2-directed antibody, and topoisomerase inhibitor drug conjugate was registered for the treatment of metastatic TNBC based on ASCEN trial [34]. Currently, there are no data concerning the effectiveness of this drug and the expression of Trop-2 (Trophoblast Cell Surface Antigen 2) in ACC of the breast. Of note, high rates of Trop-2 expression were recently confirmed in ACC of salivary gland carcinomas, which may be not organ-specific [35]. In addition, it is worth noting that the clinical benefit of multi-kinase inhibitors targeting vascular endothelial growth factor receptor (VEGFR) pathways, such as lenvatinib and axitinib, in patients with ACC of the head and neck was recently reported. TKIs biologic therapies in ACC of the breast require a designated prospective trial [36].

In the genetic landscape of ACC of the head and neck, the Notch signaling pathway and genes involved in chromatin regulation have emerged. The Notch pathway exhibits genetic alterations in about 13% of primary ACC, with NOTCH1 mutations being the most common. These mutations are even more prevalent in recurrent/metastatic ACC (40%) and have prognostic significance [37,38]. Last, a role of the chromatin state regulators pathway, including mutations in KDM6A, CREBBP, and SMARCA2, was reported in 35% of primary ACC, significantly more frequently in recurrent/metastatic ACC. Interestingly, there was a co-occurrence of mutations in NOTCH1 and chromatin remodeling genes in recurrent/metastatic ACC, suggesting a potential role of chromatin state regulators in promoting Notch signaling and ACC progression [37,39].

Another emerging therapeutic target in ACC is the MYB-NFIB gene fusion. A recent study revealed that ACC tumors, regardless of their tissue of origin, exhibit remarkably similar transcriptional profiles, which may be influenced by the activation of MYB or MYBL1 oncogenes. These gene expression patterns seem to outperform other clinical markers in the identification of high-risk ACC patients [40]. This opens the search for new targeted therapies that may improve patient outcomes.

## 5. Conclusions

In conclusion, the management of ACC of the breast, particularly in its advanced stages, poses a substantial challenge due to its rarity, diverse clinical behavior, and unpredictable response to conventional therapeutic modalities. Currently, there is no FDA-approved systemic agent in ACC of the breast. Metastasectomy is the best approach in oligometastatic disease. In turn, the best management in systemically metastasized disease is participation in clinical trials.

## Figures and Tables

**Table 1:** Immunohistochemical staining in breast ACC and kidney metastasis tissues.

	BREAST ACC	KIDNEY METASTASIS
**CYTOKERATINS**	+	+
**CK5/6**	+	+
**CK7**	+	ND
**CK18**	ND	−
**CK19**	+	+
**CK20**	ND	−
**CD7**	+/−	ND
**CD10**	−	−
**VIMENTIN**	+	+
**CD117**	+/−	+/−
**PAX-8**	−	−
**PR**	−	ND
**ER**	−	ND
**CD10**	−	−
**S100**	−	ND
**GATA-3**	−	−
**ABPAS**	ND	+
**AMACAR**	ND	−
**SMA**	+	−/+
**CEA**	ND	−
**CALPONIN**	ND	−
**P53**	ND	−
**P63**	+	ND

ABPAS—Alcian blue periodic acid-Schiff, AMACAR—Alpha methyacyl CoA racemase, CEA—Carcinoembryonic antigen, CK—cytokeratin, ER—estrogen receptor, GATA-3—GATA binding protein 3, PAX-8—paired-box gene 8, PR—progesterone receptor, SMA—smooth muscle actin, ND—not done.

**Table 2:** Details of metastatic cases of adenoid cystic carcinoma (ACC), reported in the literature.

Article	Age	Stage	Primary Treatment	DFS	Cancer Dissemination	Secondary Treatment	Treatment Response
Glover et al., 2016 [19]	50	T1N0M0	Mastectomy	13 yrs	Bones (clavicle)	metastasectomy, adjuvant RTH	-
Monga et al., 2016 [20]	57	-	Lumpectomy + RTH	8 yrs	Lung (isolated) Multiple after 3 yrs: scalp lesion, bones (rib, vertebrae, femur, pelvis)	Lobectomy next 3 years removal of skin lesion, palliative RTH (spine lesion), CTH (PXL)	OS 2 yrs
Mhamdi et al., 2017 [21]	65	T3N0M0	Mastectomy with lymphadenectomy + RTH	4 yrs	Lungs, kidney, brain, pancreas	Metastasectomy, RTH	-
Herzberg et al., 1991 [22]	57	T1cN0M0	Mastectomy	6 yrs	Lung After 12 yrs kidney	Metastasectomy	CR (2 yrs of follow-up after surgery)
Sołek et al., 2020 [23]	41	T1N0Mx	Lumpectomy + RTH	23 mo(s)	Brain, lungs, liver	CTH (4 AC cycles), metastasectomy (lungs), RTH and CTH (AC, DXL, capecitabine, and cisplatin in monotherapy)	SD
Sołek et al., 2020 [23]	52	T2N0Mx	Mastectomy + RTH	1 mo	Lungs	4 different CTH (no details)	SD
Hassoun et al., 2016 [24]	40	-	Mastectomy	15 yrs	Lungs After 10 yrs kidney	Right upper lobectomy	-
Vranic et al., 2007 [25]	71	T1cN0M0	Mastectomy	5 yrs	Kidney	Radical nephrectomy	CR (OS 12 yrs, death not related to cancer)
Nozoe et al., 2018 [26]	85	T3N0M1 (lungs)	Mastectomy, refused CTH	-	-	-	-
Silva et al., 2011 [27]	37	T2N1M0	Mastectomy + adjuvant CTH (6 AC cycles) + RTH	2 yrs	Lungs, liver, after a yr cerebellum, brainstem, bones	CTH (6 DXL + NVB (6 cycles), after a yr CTH (5-FU)	OS 3 yrs
Kim et al., 2014 [28]	33	T2N0M0	Lumpectomy + RT + adjuvant CTH (FAC)	28 mo(s)	Lungs after 17 months recurrence in lungs	Metastasectomy, palliative CTH (6 DXL cycles + 3 capecitabine cycles after recurrence)	SD (1 yr follow-up)
Kim et al., 2014 [29]	58	T2N0M0	Mastectomy with lymphadenectomy + adjuvant CTH (6 CAP cycles)	6 yrs	Lungs, bone (scapula), and liver	Palliative RTH (scapula)	PD (1 mo)
Koller et al., 1986 [30]	49	T?N0	Mastectomy	12 yrs	Lungs, brain	Metastasectomy	CR (lost to follow-up after 5 yrs)
Gillie et al., 2020 [31]	67	T2N0M0	Mastectomy with lymphadenectomy	1 yr	Liver, spleen	Best supportive care	-
Vasudevan et al., 2023 [32]	48	T1N0M0	R1 lumpectomy followed by mastectomy with lymphadenectomy + tamoxifen (5 yrs)	11 yrs	Left chest (2 lesions), After 1 yr local recurrence and progression in lungs	Metastasectomy + RTH (refused CTH)	_
Lei et al., 2023 [33]	70	T4N1M0	Mastectomy + lymphadenectomy + CTH (2 AC cycles))	1 mo	Rib, lungs	palliative CTH (10 non-specified cycles)	OS 1 yr

AC—doxorubicin + cyclophosphamide; CAP—cyclophosphamide + doxorubicin + cisplatin; CTH—chemotherapy; DFS—disease-free survival; DXL—docetaxel; FAC—5-fluorouracil + doxorubicin + cyclophosphamide; mo—month; NVB—vinorelbine; OS—overall survival; PXL—paclitaxel; RTH—radiation therapy.

## Data Availability

Additional patient data can be obtained from the authors for reasonable request.

## Abbreviations

ABPAS	Alcian Blue periodic acid-Schiff
ACC	adenoid cystic carcinoma
AMACAR	alpha-methylacyl-CoA racemase
BC	breast cancer
BLIA	basal-like immune-activated
BLIS	basal-like immune-suppressed
CAP	cyclophosphamide + doxorubicin + cisplatin schedule
CEA	carcinoembryonic antigen
CK	cytokeratin
CR	complete response
CT	computed tomography
CTH	chemotherapy
DFS	disease-free survival
DXL	docetaxel
DXR	doxorubicin
EBUS	endobronchial ultrasound
ER	estrogen receptor
FAC	5-fluorouracil + doxorubicin + cyclophosphamide schedule
GATA-3	GATA binding protein 3
HE	hematoxylin and eosin
HER2	human epidermal growth factor receptor 2
LAR	luminal androgen receptor
MES	mesenchymal
mo	month
ND	not done
NVB	vinorelbine
OS	overall survival
PAX-8	paired-box gene 8
PD	progressive disease
PET-CT	positron emission tomography-computed tomography
PR	progesterone receptor
RTH	radiation therapy
SD	stable disease
SMA	smooth muscle actin
SLNB	sentinel node biopsy
TKI	tyrosine kinase inhibitor
